# Automated Multiclass Artifact Detection in Diffusion MRI Volumes *via* 3D Residual Squeeze-and-Excitation Convolutional Neural Networks

**DOI:** 10.3389/fnhum.2022.877326

**Published:** 2022-03-30

**Authors:** Nabil Ettehadi, Pratik Kashyap, Xuzhe Zhang, Yun Wang, David Semanek, Karan Desai, Jia Guo, Jonathan Posner, Andrew F. Laine

**Affiliations:** ^1^Heffner Biomedical Imaging Laboratory, Department of Biomedical Engineering, Columbia University, New York, NY, United States; ^2^Department of Psychiatry and Behavioral Sciences, Duke University, Durham, NC, United States; ^3^Department of Psychiatry, Columbia University Medical Center, New York, NY, United States; ^4^Zuckerman Institute, Columbia University, New York, NY, United States

**Keywords:** diffusion MRI, artifacts, quality control, convolutional neural networks, deep learning, medical imaging

## Abstract

Diffusion MRI (dMRI) is widely used to investigate neuronal and structural development of brain. dMRI data is often contaminated with various types of artifacts. Hence, artifact type identification in dMRI volumes is an essential pre-processing step prior to carrying out any further analysis. Manual artifact identification amongst a large pool of dMRI data is a highly labor-intensive task. Previous attempts at automating this process are often limited to a binary classification (“poor” vs. “good” quality) of the dMRI volumes or focus on detecting a single type of artifact (e.g., motion, Eddy currents, etc.). In this work, we propose a deep learning-based automated multiclass artifact classifier for dMRI volumes. Our proposed framework operates in 2 steps. In the first step, the model predicts labels associated with 3D mutually exclusive collectively exhaustive (MECE) sub-volumes or “slabs” extracted from whole dMRI volumes. In the second step, through a voting process, the model outputs the artifact class present in the whole volume under investigation. We used two different datasets for training and evaluating our model. Specifically, we utilized 2,494 poor-quality dMRI volumes from the Adolescent Brain Cognitive Development (ABCD) and 4,226 from the Healthy Brain Network (HBN) dataset. Our results demonstrate accurate multiclass volume-level main artifact type prediction with 96.61 and 97.52% average accuracies on the ABCD and HBN test sets, respectively. Finally, in order to demonstrate the effectiveness of the proposed framework in dMRI pre-processing pipelines, we conducted a proof-of-concept dMRI analysis exploring the relationship between whole-brain fractional anisotropy (FA) and participant age, to test whether the use of our model improves the brain-age association.

## Introduction

Diffusion weighted imaging (DWI; Stejskal and Tanner, 1965; Le Bihan et al., 1986; Huisman, 2003; Baliyan et al., 2016), as well as diffusion tensor imaging (DTI; Basser and Jones, 2002; Alexander et al., 2007), are widely used these days in brain research and clinical neuroimaging (Huisman, 2010). Using dMRI one is able to gain insight into white matter’s development based on the different diffusion rates of water across different brain tissues (Hüppi and Dubois, 2006; Ladouceur et al., 2012; Simmonds et al., 2014). Moreover, dMRI provides means for investigation of other abnormal white matter developments such as Schizophrenia (Roalf et al., 2015; Tønnesen et al., 2018) and Alzheimer’s disease (Hoy et al., 2017; Lo Buono et al., 2020). dMRI data can also be used for different brain biomarker measurements in large population studies such as the Human Connectome Project (Van Essen et al., 2012). However, dMRI data is often contaminated by various sources of artifacts such as motion, Eddy currents, low signal to noise ratio (SNR), gradient distortions, chemical shift, susceptibility, Gibbs ringing, etc., (Le Bihan et al., 2006; Krupa and Bekiesińska-Figatowska, 2015). Existence of such artifacts in dMRI volumes without any exclusion or correction could bias the results of any subsequent analysis and make their interpretation unreliable (Bammer et al., 2003; Van Dijk et al., 2012; Reuter et al., 2015). Hence quality control and artifact identification in dMRI data is an essential pre-processing step before conducting any analysis.

Current approaches for quality control and artifact identification in dMRI data are mostly performed manually by visual inspection of all volumes (sometimes even all slices) by an expert(s). This process is extremely labor-intensive and suffers from being subjective in nature. Hence there is a need for automated ways of artifact identification that are fast and reliable.

Computerized approaches for quality control and artifact identification of dMRI data are able to alleviate the challenge of manual inspection. Throughout the years, several tools for automated quality control of dMRI data have been proposed such as FSL (Jenkinson et al., 2012; Andersson et al., 2016; Bastiani et al., 2019), DTIPrep (Oguz et al., 2014), DTI studio (Jiang et al., 2006), and TORTOISE (Pierpaoli et al., 2010). More recently, various statistical (Roalf et al., 2016) or Artificial Intelligence (AI) approaches for quality control and artifact detection of dMRI data have been introduced (Iglesias et al., 2017; Kelly et al., 2017; Alfaro-Almagro et al., 2018; Fantini et al., 2018; Graham et al., 2018; Samani et al., 2020; Ahmad et al., 2021; Ettehadi et al., 2021). However, most of these approaches either only operate on a binary-level (i.e., distinction of “poor-quality” data from “good-quality” without identifying the specific artifact type) (Samani et al., 2020; Ahmad et al., 2021; Ettehadi et al., 2021), or have been designed to only detect a single specific type of artifact (e.g., motion) (Iglesias et al., 2017; Kelly et al., 2017; Fantini et al., 2018). A detailed report on the performance of such tools can be found in Liu et al. (2015), Haddad et al. (2019).

In this work, we propose a deep learning-based framework for automatic multiclass artifact classification in poor-quality dMRI volumes. Unlike previous work, our framework is not customized for a single specific artifact type and takes into account a wider range of artifacts to classify. In particular, our method classifies four classes of artifacts namely: motion, out of field of view (FOV), low signal to noise ratio (SNR), and MRI miscellaneous artifacts. The MRI miscellaneous artifacts category serves as a control group for the classifier which includes other MRI (as well as dMRI) artifacts that do not necessarily belong to any of the other three classes.

The proposed method operates in two steps. First, the dMRI volumes are partitioned into MECE slabs and fed to a designed convolutional neural network (CNN). The designed CNN then outputs the artifact class labels of the slabs. Second, through a voting process, the slab-level predicted labels are utilized to decide on the final label for the whole dMRI volume (i.e., volume-level artifact label). We tested our method on two separate datasets. Our results demonstrate that the proposed framework can be utilized for fast automatic classification of the four categories of artifacts considered here. Moreover, the extended validation analysis calibrating FA-age correlation demonstrates an improvement using the models’ predicted artifact labels. These results together suggest that the model can be utilized in dMRI pre-processing pipelines to improve the results of subsequent analyses.

## Materials and Methods

**FIGURE 1:**
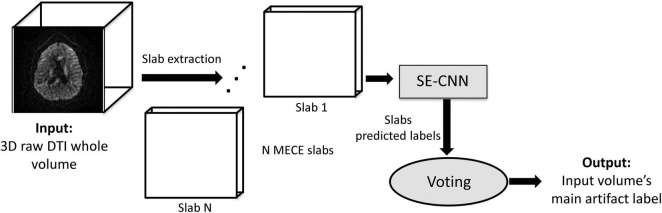
A schematic overview of the proposed multiclass volumetric dMRI artifact classifier.

As mentioned in the introduction section, the proposed framework employs a 2-step approach to classify poor-quality dMRI volumes into four categories of artifacts. In the first step, the prominent artifact class labels of individual MECE 3D slabs (extracted from dMRI whole volumes) are predicted through design and training of a residual squeeze and excitation (SE) CNN. In the second step, the most consistent label amongst the predicted slabs’ labels is chosen *via* a voting system as the prominent artifact type present in the whole dMRI volume. Figure 1 shows an overview of the multiclass major artifact detection framework. As a proof of concept, we utilize the labels generated by the framework to run an FA-age correlation analysis in order to test the model’s efficacy. In this section, details of our 2-step approach, the conducted FA-age analysis, and the datasets used in this work are discussed.

### Slab-Level Artifact Classification Using Residual SE-CNN

Due to memory limitations of the currently available typical GPUs, it is often not feasible to feed a whole 3D dMRI volume into a CNN. Hence, images need to be partitioned prior to feeding them to a GPU. Therefore, to address this issue while capturing artifact patterns over the entire 3D image without missing any region, we partition the dMRI volumes into a number of MECE slabs spanning the entire volume at hand. In addition to addressing GPU memory limitations, the MECE slab-based classifier approach has two benefits: (a) it allows the proposed residual SE-CNN model to capture local information, and to achieve global consistency, by sweeping the entire volume through 3D MECE regions, and (b) it allows utilization of a voting approach to predict the artifact type of a whole dMRI volume, thereby making the final prediction more robust to slab-level misclassifications.

**FIGURE 2:**
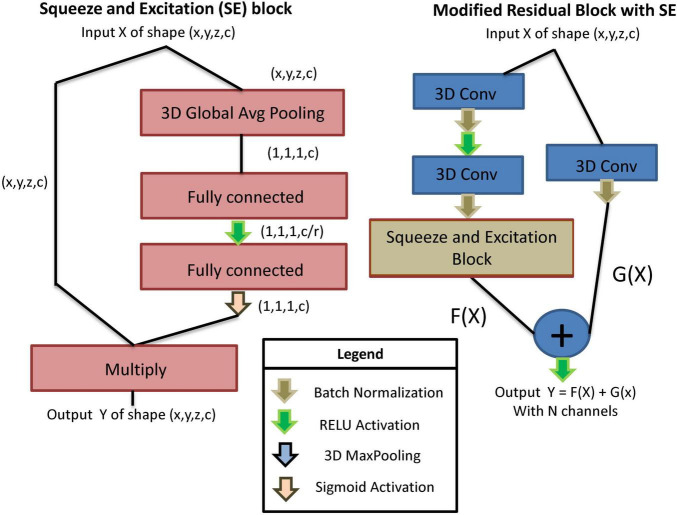
Left: The building blocks of an SE block. Right: A modified residual block with SE. The double convolutional path of the residual block is equipped with a SE block that captures attention weights for different channels of the convolutional kernels.

In order to classify the main artifact type in each slab associated with a dMRI volume, we use a custom-designed residual SE-CNN architecture. Our custom residual SE-CNN architecture consists of several cascaded modified versions of original residual blocks (He et al., 2016) equipped with squeeze and excitation components (Hu et al., 2018). The SE block is shown in Figure 2 (left), and the modified residual block with SE is depicted in Figure 2 (right). The SE block is a self-attention unit that aims at modeling the interdependencies between different channels within the layers (Hu et al., 2018). Essentially, SE blocks learn how to filter information across channels, in order to focus on the most relevant features for the classification task. This is achieved through learning the relative importance weights of different channels. The process of learning the channel weights is done *via* a global average pooling along the image spatial dimensions (i.e., x, y, and z) followed by two fully connected layers with non-linear activation functions operating on the channel dimension. The key hyperparameter of a SE block is the squeeze parameter r (in the first fully connected layer) that reduces the number of channels by a ratio of r to find the most important channel representation for the classification task. After learning the channel weights, they are projected onto the original feature tensor by element-wise multiplication (see Figure 2, left). For more information on this topic, readers are directed to Hu et al. (2018). The SE block is then placed in the double convolutional path of the modified residual block to learn the channels weights of the two preceding 3D convolutional kernels (see Figure 2, right). This block is called modified residual block with SE and is used sequentially in our classifier’s architecture.

**FIGURE 3:**
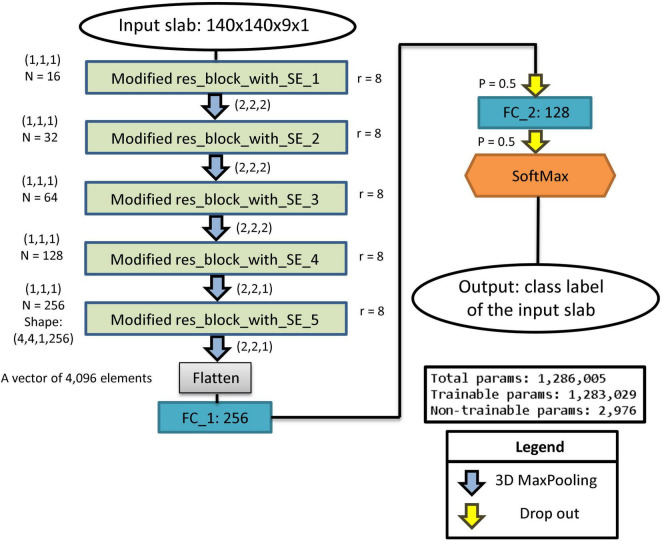
The architecture of the residual SE-CNN slab-level artifact classifier.

The architecture of the proposed residual SE-CNN artifact classifier is shown in Figure 3. As depicted in Figure 3, our residual SE-CNN model is built with five stacked modified residual blocks with SE. After each block, a 3D Maxpooling layer is used to reduce the image dimensions. Throughout the network, the image spatial dimension is reduced while the number of channels is increased. For each layer, the kernel sizes and the number of channels are presented in Figure 3. After the 5th modified residual block with SE, the features vector (consisting of 4,096 elements) is unrolled and fed through two consecutive fully connected layers. Finally, a SoftMax classifier with 4 possible classes (motion, out of FOV, low SNR, and MRI miscellaneous artifacts) is used to perform the slab-level main artifact type classification. To regularize the model and minimize overfitting effects dropout units are placed after the fully connected layers.

### Whole-Volume Artifact Prediction

After predicting the slab-level labels *via* the residual SE-CNN model, we utilize these labels to predict the main artifact label associated with the whole 3D dMRI volume *via* the voting block (Figure 1). The voting block takes the predicted labels for the N MECE slabs of a dMRI whole volume as inputs and outputs the main existing artifact type of that dMRI volume. This is achieved *via* maximum consensus on the predicted labels between the slabs as detailed by Algorithm 1 presented in Table 1.

**TABLE 1:** Detailed steps of Algorithm 1 for voting on the final labels of dMRI whole volumes.

Algorithm 1: Generating whole volume artifact labels
**Input:** N slabs’ predicted labels: *L* = {*l*_1_,*l*_2_,…,*l*_*N*_} **Output:** Whole volume’s predicted artifact label: *L*_*Vol*_ **Procedure:** - Let integers 0, 1, 2, and 3 denote the four possible values for each label *l_i_* - Let *n_j_* denote the count for the *j^th^* possible label: ● Then for each vector *L* we have: *N* = {*n*_0_,*n*_1_,*n*_2_,*n*_3_} - The whole volume label is predicted as follows: ● *L*_*Vol*_ = *argmax*{*n*_*j*_} for j = 0, 1, 2, and 3

### Fractional Anisotropy-Age Analysis as a Proof of Concept to Demonstrate Efficacy of the Artifact Classifier

The 4,226 manually labeled poor-quality dMRI volumes of the HBN dataset (details of this dataset are presented in the data and pre-processing section) were derived from 66 randomly selected subjects from a larger pool of 100 participants whose age varied from 5.57 to 21.89 years with mean and median age of 10.95 and 10.05 years, respectively. The HBN dataset was selected because the age range was appropriate for the desired age-based analysis, and because many of the MRI scans were known to be corrupted with artifacts based on our prior experience with this dataset (Luna et al., 2021). Prior research has demonstrated a linear relationship between participant age and FA (Rathee et al., 2016; Richie-Halford et al., 2021). Exploiting this fact, a proof-of-concept analysis examined the bivariate correlation between mean whole-brain FA and participant age.

Two tests (referred to as A and B) were conducted in which A: the age of 100 HBN participants was correlated with their mean whole-brain FA values and, B: the same analysis was re-run after removing artifact corrupted dMRI volumes (as labeled by the SE-CNN artifact classifier). Following similar methods described elsewhere [e.g., (Pujol et al., 2016; Chow and Chang, 2017)], scans had their corrupted volumes removed if the number of poor-quality dMRI volumes were between 1 and 26 (or <20% of the dMRI volumes). Of note, scans with >26 poor-quality dMRI volumes were not excluded from this analysis to ensure the same sample size across the A/B testing. Of the 66 scans, the dMRI data of 17 were affected by the A/B testing. An increase in the FA-age correlation will support the efficacy of the classifier.

### Data and Pre-processing

Diffusion MRI data is highly heterogeneous due to various reasons such as scanner differences, diffusion directions (various gradient directions), demographics (e.g., age, gender, etc.), etc., (Ahmad et al., 2021). This heterogeneity makes it almost impossible to train a CNN on a dataset and use the trained model (without changing the learned parameter values) as is to account for the differences that may be found while analyzing a different study (Ahmad et al., 2021). Hence, to demonstrate the feasibility of high accuracy automated artifact classification using the proposed residual SE-CNN architecture, we trained our model on two different datasets separately, and evaluated the results. Namely, we used the Adolescent Brain Cognitive Development (ABCD; Casey et al., 2018) and Healthy Brain Network (HBN; Alexander et al., 2017) datasets. By training the model separately for each dataset, the model’s parameters are learned optimally according to the target distribution. In what follows we discuss the two datasets as well as the annotation and pre-processing steps.

#### Adolescent Brain Cognitive Development Dataset

The goal of the ABCD study is to track human brain development over time (childhood through adolescence) (Casey et al., 2018). For this purpose, the study hired more than 10,000 participants between the age of 9–10 years old. Institutional review boards at 21 different sites that were involved in this study approved the study protocols. The ABCD dataset is available at^1^. In this work we utilized multi-shell (*b* = 0, 500, 1000, 2000, 3000 s/mm2) diffusion scans from 85 participants. All scans are isotropic [1.7 × 1.7 × 1.7 mm3 with matrix size of (140 × 140 × 81)] and have identical diffusion directions (96). Due to imaging across 21 different sites, the acquisition parameters are slightly different which aids the model to learn robustness to heterogeny of the acquisition parameters within the dataset.

#### Healthy Brain Network Dataset

The Child Mind Institute^2^ launched the HBN study in 2017 (Alexander et al., 2017) and made the data publicly available at^3^. This ongoing study focused on creating a large-scale dataset of 10,000 5–21 years old New York City area children and adolescents. This study utilized a community-referred recruitment strategy. The study design was approved by the Chesapeake Institutional Review Board^4^. In this work, we used multi-shell (*b* = 0, 1000, 2000 s/mm2) dMRI scans from 100 distinct subjects. All scans have isotropic resolution (1.8 × 1.8 × 1.8 mm3), with 72 slices, and identical diffusion directions (64). Since the in-plane matrix size of HBN dataset (mostly 104 × 104) is different from that of ABCD (i.e., 140 × 140) we resized the HBN images in the xy plane using the Bi-cubic interpolation implemented in the scikit-image Python library (Van der Walt et al., 2014). The resized HBN volumes have a matrix size of (140 × 140 × 72).

#### Manual Annotation and Class-Distribution

**FIGURE 4:**
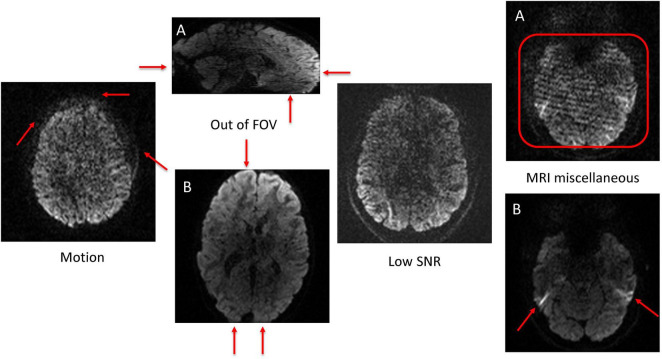
Examples of the four artifact classes. Artifact patterns are demonstrated with red arrows or rectangles. The motion artifact blurring patterns are observable across the border between the brain and the background as depicted by the red arrows. To better observe the out of FOV class, two views of the same volume were depicted [**(A)**: Sagittal and **(B)**: Axial]. The example for the low SNR class, shows a poor signal to noise ratio all over the image. Two different artifact types (form two different volumes) belonging to the MRI miscellaneous artifact class are shown in the right side of the figure [**(A)**: Herringbone style artifacts are evident in the region shown by the red rectangle; **(B)**: Susceptibility artifact are show on both sides of the brain by the red arrows].

**FIGURE 5:**
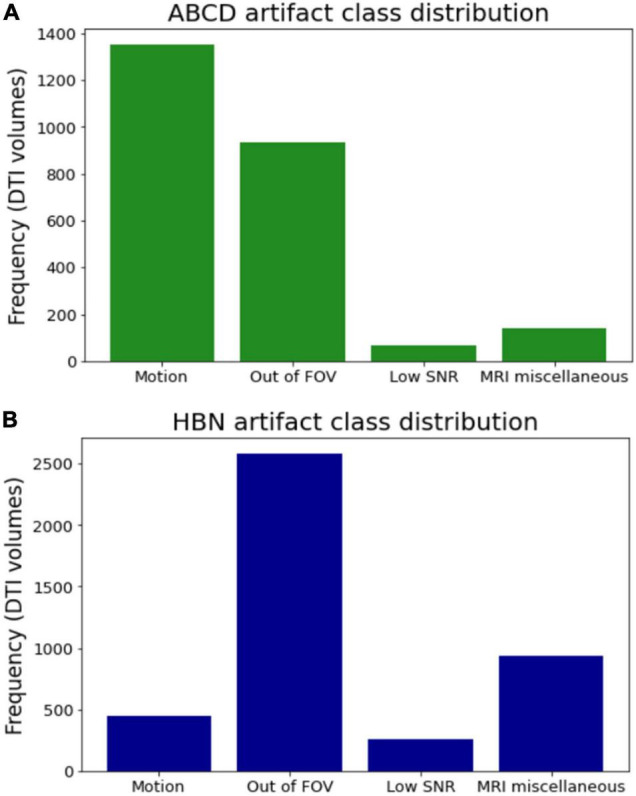
Class distribution for the two datasets. **(A)** The ABCD dataset has 1,353 volumes contaminated with motion, 933 volumes being out of FOV, 67 volumes with low SNR, and 141 volumes with MRI miscellaneous artifacts. **(B)** The HBN dataset contains 449 volumes with motion, 2,583 volumes being out of FOV, 258 volumes with low SNR, and 936 volumes with MRI miscellaneous artifacts.

All clean (no artifacts) volumes in both ABCD and HBN datasets were excluded from this work (except for the FA-age analysis). The remaining poor-quality volumes in both datasets (2,494 volumes in ABCD and 4,226 volumes in HBN) were manually annotated (at volume-level not slice-level, this reduces the labor intensity of manual annotation) into four main artifact classes (indexed as: 0: motion, 1: out of FOV, 2: low SNR, and 3: MRI miscellaneous artifacts) by an expert with 12 years of experience in MRI and DTI analysis. The MRI miscellaneous artifacts class refers to all other types of MRI (and dMRI) artifacts (such as Eddy currents, ghosting, etc.) that do not necessarily fall into the other three artifact categories considered here. One of the reasons for having this broad class is due to the difficulty of identifying a single major source of artifact in some dMRI volumes. Hence by having this class, the model can learn to distinguish between the other three prominent artifact types and classify a poor-quality volume into a 4th class if it doesn’t fall into the other three categories. Figure 4 illustrates examples of the artifact classes considered in this work. The volume-wise manually annotated class distributions in ABCD and HBN datasets are shown in Figure 5. The labeled volumes are randomly assigned to train, validation, and test sets using a split ratio of 6:2:2. The intensity of each volume is normalized to the range of (0, 1). To capture the visual patterns of the artifacts that manifest themselves better in the border between the brain and background (such as motion and out of FOV), no brain extraction or background removal was carried out.

#### Fractional Anisotropy-Age Pre-processing Pipeline

Standard dMRI pre-processing using MRtrix (Tournier et al., 2019) and FSL (Jenkinson et al., 2012) including denoising, EDDY (Andersson and Sotiropoulos, 2016), TOPUP (Andersson et al., 2003), and bias field correction (Zhang et al., 2001) was carried out for all 100 HBN participants followed by derivation of whole-brain FA values (test A). The same pipeline was executed in addition to removing some of the noisy dMRI volumes (B). It is to be noted that the pre-processing can clean the dMRI data, but this cleaning is consistent across A/B.

## Results

In this section we discuss our implementation and the results. In particular, we detail the slab extraction procedure, the architecture’s hyperparameters, as well as the choice of hyperparameters for training the residual SE-CNN artifact classifier. Next, we present the slab-level classification results of the residual SE-CNN classifier on the ABCD and HBN test sets as well as the whole volume prediction accuracy using the voting procedure. Finally, we discuss the results of the FA-age analysis on the HBN dataset.

### Extraction of Mutually Exclusive Collectively Exhaustive Slabs From Whole Volumes

As mentioned in the Materials and Methods section, we partition a whole dMRI volume into *N* MECE 3D slabs. Concatenation of these slabs along the *z* dimension forms the original dMRI volume without any loss of information. We chose a slab size of (140, 140, 9). Using this slab size, we end up with nine slabs per each dMRI volume in the ABCD and eight slabs for each dMRI volume in the HBN dataset. The slabs are then fed to the residual SE-CNN as inputs.

### Model Hyperparameters and Training History

The architecture of our proposed residual SE-CNN was presented in Figure 3. This architecture with its set of chosen hyperparameters was used for both ABCD and HBN datasets. In the implementation phase, for all convolutional layers, RELU activation function was used (if any) as the choice of non-linearity unless explicitly noted. The kernels’ parameters were initialized *via* the random Glorot initialization technique (Glorot and Bengio, 2010). All convolutional kernel sizes were set to (1, 1, 1) with zero paddings to keep the spatial dimensions same before and after employment of the convolutional filters. For the Max-pooling layers, the first three consecutive blocks, used a pooling size of (2, 2, 2), and the pooling size of (2, 2, 1) was used for the last two blocks. The squeeze parameter *r* was set to 8. We used the RELU activation function for the last two fully connected layers (with 256 and 128 nodes, respectively). The probability for the two dropout units was set to 0.5. Finally, we used a SoftMax activation function for the SoftMax layer with four artifact classes: 0 (motion), 1 (out of FOV), 2 (low SNR), and 3 (MRI miscellaneous artifacts). The architecture was implemented in Python, using Keras with TensorFlow as backend. Except for the number of epochs, the training hyperparameters were also the same for both datasets. The model was trained for 2000 epochs on the ABCD dataset while the HBN dataset converged slightly faster and was trained for 1500 epochs. The batch size was set to 32. The model was trained, separately for ABCD and HBN, to minimize the categorical cross-entropy loss function using the manually labeled data in their respective training sets. For optimizing the cost function, SGD with Nesterov optimizer (Sutskever et al., 2013) (initial learning rate = 0.0001, Momentum = 0.6, and decay rate = 10^–6^) was used. A summary of the hyperparameters and their values are presented in Table 2. In what follows we discuss the results on the two datasets separately.

**TABLE 2:** The model and training’s main hyperparameters.

Hyperparameter	Value/Algorithm
Kernel initialization technique	Glorot
Convolutional kernel size	[1,1,1] With zero paddings
Maxpooling kernel size	[2,2,2] (Last two layers: [2,2,1])
Squeeze parameter *r*	8
Activation function	RELU (Unless otherwise mentioned)
Dropout probability	0.5
Batch size	32
Optimizer	SGD with Nesterov Initial learning rate = 0.0001 Momentum = 0.6 Decay rate = 10^–6^
Loss function	Categorical cross-entropy

### Evaluation on the Adolescent Brain Cognitive Development Dataset

**FIGURE 6:**
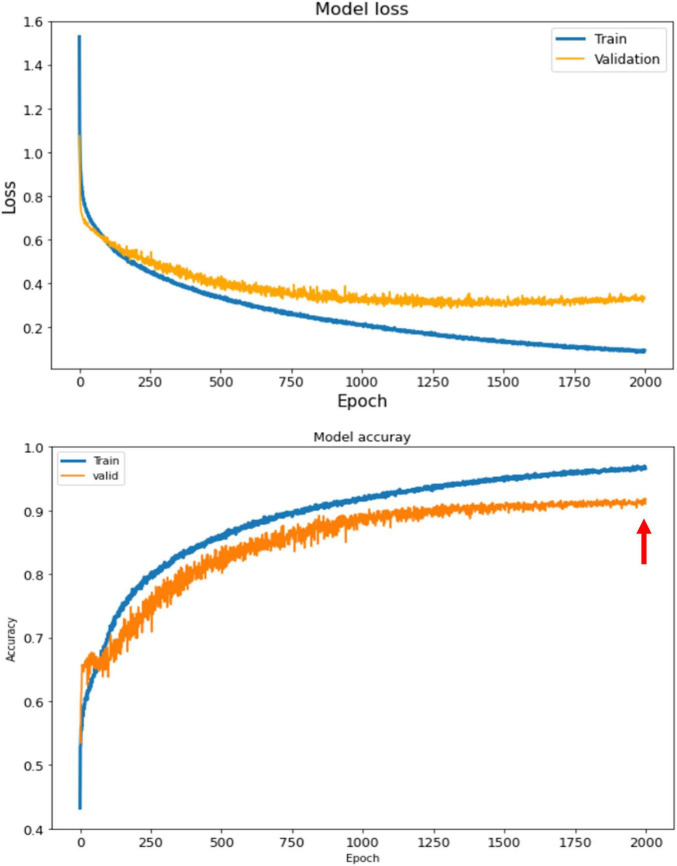
The training history for ABCD dataset. The loss (top) and the accuracy (bottom) for train and validation sets. The best performing model is the model with highest accuracy on the validation set which happens at epoch 1999 shown by the red arrow.

**FIGURE 7:**
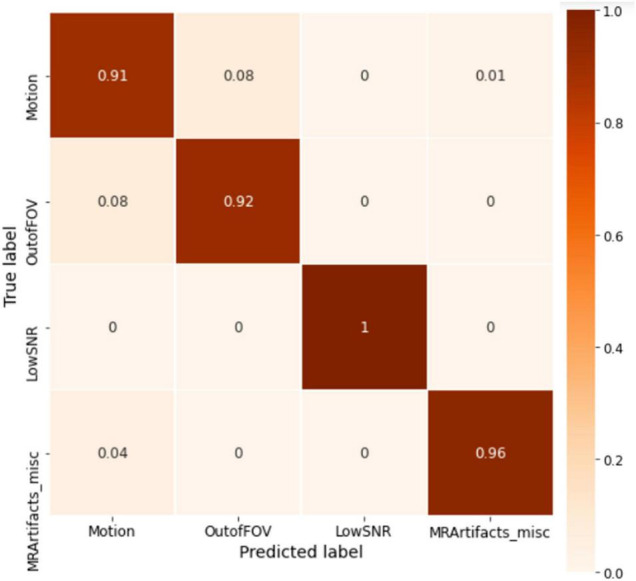
The slab-level confusion matrix for the ABCD test set. The matrix shows roughly a diagonal behavior with minor misclassifications between motion and out of FOV classes.

**FIGURE 8:**
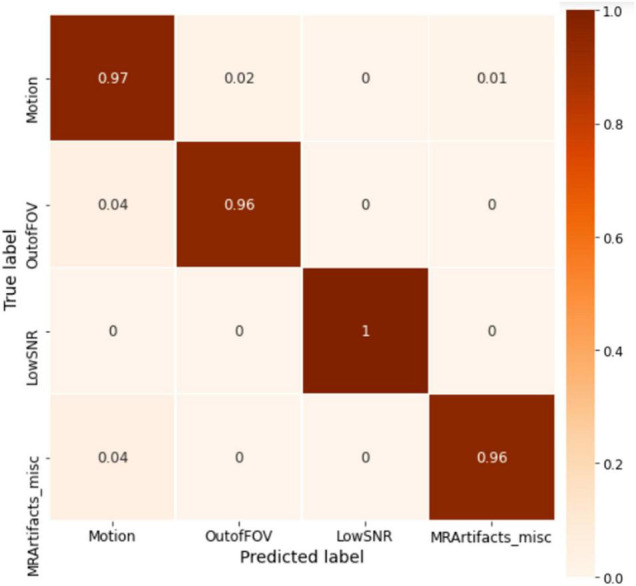
The whole volume-level confusion matrix for the ABCD test set. After utilizing the voting process, the matrix turns more diagonal as the misclassification errors get smaller.

The ABCD training history is depicted in Figure 6. As depicted, the training is stable and the overall accuracy is increasing over epochs. The criterion used for model selection was the highest overall classification accuracy on the validation set. The test set’s slab-level confusion matrix for the best performing model (at epoch 1999) is presented in Figure 7. The slab-level classification accuracy of the model on the training, validation, and test sets were 96.80, 92.00, and 91.86%, respectively. Using the voting procedure discussed in Algorithm 1, we evaluated the performance of the model on whole volume major artifact classification. The model achieved a test set primary artifact classification average accuracy of 96.61%. The confusion matrix for whole volume predictions is depicted in Figure 8.

### Evaluation on the Healthy Brain Network Dataset

**FIGURE 9:**
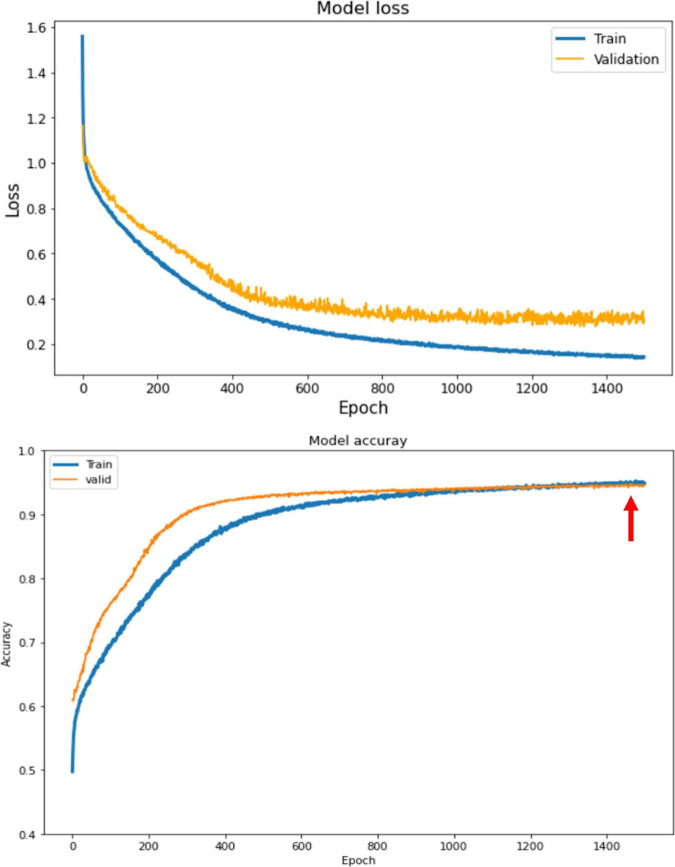
The training history for HBN dataset. The loss (top) and the accuracy (bottom) for train and validation sets. The highest slab-level validation accuracy model happens at epoch 1462 and is shown by the red arrow.

**FIGURE 10:**
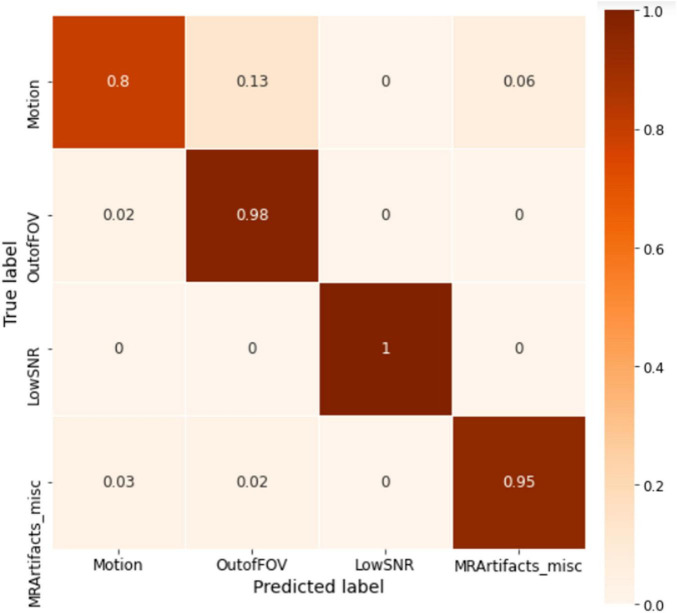
The slab-level confusion matrix for the HBN test set. The matrix is mostly diagonal with some misclassifications in the motion class.

**FIGURE 11:**
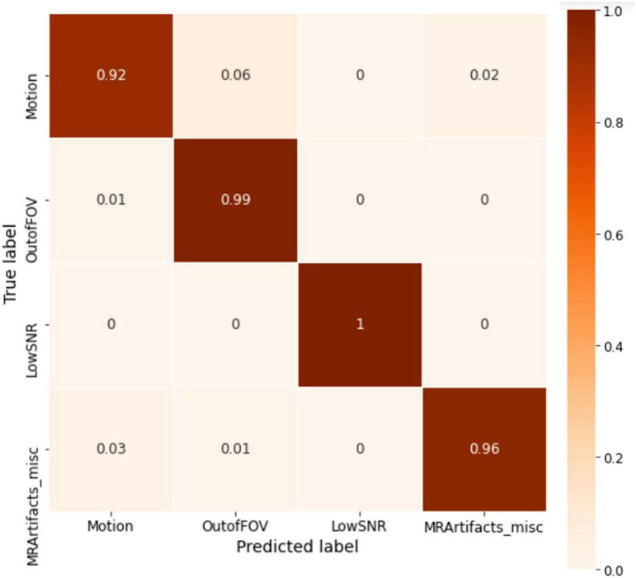
The whole volume-level confusion matrix of the test set of HBN. Voting process further decreased the misclassifications and turned the matrix more diagonal.

The model was trained for 1500 epochs (using the exact same training hyperparameters as in ABCD) on the HBN dataset and the training history is shown in Figure 9. Similar to the ABCD, the training process also shows a stable behavior for the HBN dataset. The highest validation accuracy model (*i.e.*, the best model) was achieved at epoch 1462 resulting in slab-level classification accuracies of 95.00, 94.69, and 95.77% on the train, validation, and test sets, respectively. Figure 10 shows the slab-level artifact classification confusion matrix. Although the accuracy on the motion class is below 90%, through the voting process this accuracy increases as the misclassification errors become smaller by voting. To demonstrate this, Figure 11 shows the volume-level classification confusion matrix. The model archived an overall test set volume-level accuracy of 97.52% and the accuracy on the motion class improved significantly.

### Fractional Anisotropy-Age Analysis Results

**FIGURE 12:**
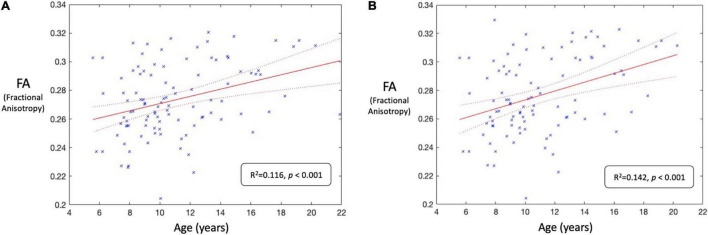
**(A)** Correlation analysis between whole-brain fractional anisotropy (FA) and age (in years) for test A (i.e., all 100 HBN participants). The solid red line denotes the regression line, and the two curved red dashed lines denote the 95% confidence intervals. **(B)** Correlation analysis between whole-brain fractional anisotropy (FA) and age (in years) for test B (i.e., 100 HBN participants with some noisy (labeled by SE-CNN classifier) dMRI volumes removed). The solid red line denotes the regression line, and the two curved red dashed lines denote the 95% confidence intervals. The FA-age relationship improved after utilization of the labels generated by the model to stratify the data.

Results of the A/B testing demonstrate an improvement in FA-age correlation brought about by including the labels from the residual SE-CNN model. Figures 12A,B depict the linear regression fit with respective 95% confidence intervals for the FA-age relationship for the tests A and B, respectively. Test A demonstrates an *R*^2^ of 0.116 whereas test B demonstrates an *R*^2^ of 0.142 at the same significance level (*p* < 0.001).

## Discussion

### Summary, Strengths, and Shortcomings

In this work, we developed an automatic 4-class major artifact classifier for 3D diffusion MRI volumes. We trained the exact same architecture with the same exact choice of hyperparameters (both model’s and training’s hyperparameters) on two different datasets, namely ABCD and HBN. Our results demonstrate the capability of this architecture and the proposed hyperparameter choices, followed by the voting procedure to accurately classify four categories of artifacts in 3D dMRI volumes.

As mentioned earlier, diffusion MRI is highly diverse in nature. In particular, different diffusion directions across various studies is an important source of heterogeneity. This makes it highly challenging to propose and train an architecture that generalizes with an acceptable accuracy to unobserved and different distributions from the one it has been trained on. One main reason for this is due to the fact that the 3D convolutional kernels, when trained on a dataset with a specific set of diffusion directions, learn their spatial parameters’ optimal values according to those specific directions. Hence, when the model faces a new dataset with different diffusion directions, the exact same learned values for the convolutional kernels are not optimal according to the new diffusion directions. Moreover, in the case of our architecture, the channel attention weights learned in the SE blocks are also learned according to the diffusion directions of the original dataset they were trained on. These learned attention weights are not the optimal values for a dataset with different diffusion directions. Hence applying a model that is trained on a dataset, while fixing all of its learned parameters’ values, to perform artifact classification on a different dataset is not a suitable solution.

To alleviate this issue, we proposed an architecture with a specific set of model’s hyperparameters (not learnable parameters, it is important to note this distinction) as well as training’s hyperparameters. We provided evidence that this architecture can be trained on a small subset of data when facing a new dataset to find the optimal kernel parameters values according to different heterogeneity sources, especially the diffusion directions. Previous works on binary (“poor” vs. “good”) quality control of dMRI volumes such as (Ahmad et al., 2021), which is a much simpler task than a 4-class artifact classification, have also proposed to re-train the network on a small subset of manually annotated data to solve the generalization issue when facing a new diffusion MRI dataset.

The major challenges with training a neural network model are twofolds: (1) the labor-intensive task of manual annotation, and (2) model’s as well as training’s hyperparameters tuning process. Through our proposed framework we have tried to address these two challenges to make it more convenient for potential users to be able to train the model according to their target diffusion MRI dataset. First, since our framework utilizes a 3D architecture, manual annotation needs to be done only at the volume level (not slice/voxel level) by assigning a single label to a whole volume. This significantly reduces the load of manual annotation task. For example, in our case, it only took our expert ∼17 min to manually annotate one subject (∼110 volumes) with four classes of artifacts. Second, we found a set of hyperparameters for the model’s architecture as well as the training’s hyperparameters, which gave us comparable results in two different datasets. Based on these results, we believe these sets of hyperparameters can be used to train the proposed architecture on a different dataset or at least serve as a starting point to reduce the search space.

As mentioned in the introduction section, most works on automated artifact detection or quality control of dMRI scans have been done on a binary (“poor” vs. “good”) level. To the best of our knowledge, our proposed framework is the first automated and accurate method to consider a wider range of artifact classification (i.e., four classes of motion, out of FOV, low SNR, and MRI miscellaneous artifacts). Collecting dMRI scans is labor intensive and a costly process, especially when dealing with infants. Hence, we believe by teasing out the type of artifact in poor-quality volumes, one might be able to correct for the detected artifact (if possible) and restore the volumes to reduce the cost of acquiring dMRI scans.

The proposed model improved in the FA-age correlation when its predicted labels were incorporated to stratify the dataset. This improvement supports the utility of the model in a standard dMRI pre-processing application. The increase in FA-age correlation after removal of corrupted dMRI volumes underscores the applicability of this artifact classifier.

While we demonstrated the capability of our method to provide a convenient mean for accurate automatic classification of four categories of artifacts in poor-quality diffusion MRI volumes, we are aware of its shortcomings. The current method only addresses four categories of artifacts. In the future we plan to further improve upon the framework to be able to consider a wider range of artifacts. In other words, we plan to further tease out the types of artifacts in the MRI miscellaneous category (the 4th class). Additionally, the current framework only identifies a single major artifact type for each volume. In the future, we plan to adapt our model to output multiple potential artifact types (if any) in cases where there might be several different artifact categories present in the volume under investigation. Moreover, the proposed method currently predicts artifact types on whole volume-level. While in most cases, the entire poor-quality volume is affected by the detected artifact and hence discarded (or corrected, if possible), in some cases where the size of the dataset is small, users are interested in only discarding (or correcting, if possible) the few slices that were affected by the detected artifact. Hence, in the future we plan to find ways and adapt our framework to further improve the resolution of our artifact classification from volume-level to slice-level.

## Conclusion

Artifact type identification is an exhaustive but essential step in pre-processing of dMRI data to discard or correct (if possible) the contaminated volumes. Without artifact correction or removal of the contaminated volumes, one cannot guarantee the accuracy of any subsequent analysis and draw reliable conclusions. In this paper, we proposed a deep learning architecture, namely a 3D residual SE-CNN, followed by a voting procedure to automatically classify poor-quality volumes into 4 categories of artifacts (i.e., motion, out of FOV, low SNR, and MRI miscellaneous artifacts). Our results demonstrate the capability of the proposed framework in accurate multiclass artifact classification. Moreover, to take into account, the heterogeneity of the dMRI data, we found a set of hyperparameters for the model as well as training hyperparameters that were able to provide accurate classifications on two different datasets (i.e., 96.61 % on ABCD’s test set and 97.52% on HBN’s). The provided sets of hyperparameters can be used to guide the potential users with training the proposed architecture according to their own dataset to compensate for the heterogeneity. This potentially enables the framework to be integrated in dMRI processing pipelines for fast automatic artifact type identification.

## Data Availability Statement

Publicly available datasets were analyzed in this study. This data can be found here: ABCD dataset: https://nda.nih.gov/abcd, and HBN dataset: http://fcon_1000.projects.nitrc.org/indi/cmi_healthy_brain_network/.

## Ethics Statement

The studies involving human participants were reviewed and approved by for ABCD dataset: Institutional review boards at 21 different sites that were involved in this study, and for HBN dataset: The Chesapeake Institutional Review Board. Written informed consent to participate in this study was provided by the participants’ legal guardian/next of kin.

## Author Contributions

NE: methodology, implementation, visualization, and writing (original draft, review and editing). PK and KD: dMRI analysis, visualization, and writing (review and editing). XZ: implementation and writing (review and editing). YW and JG: writing (review and editing). DS: data visualization, manual labeling, and writing (review and editing). JP: conceptualization, dMRI supervision, and writing (review and editing). AL: conceptualization, writing (review and editing), and supervision. All authors contributed to the article and approved the submitted version.

## Conflict of Interest

The authors declare that the research was conducted in the absence of any commercial or financial relationships that could be construed as a potential conflict of interest.

## Publisher’s Note

All claims expressed in this article are solely those of the authors and do not necessarily represent those of their affiliated organizations, or those of the publisher, the editors and the reviewers. Any product that may be evaluated in this article, or claim that may be made by its manufacturer, is not guaranteed or endorsed by the publisher.

## Footnotes

1
https://nda.nih.gov/abcd

2
https://childmind.org/

3
http://fcon_1000.projects.nitrc.org/indi/cmi_healthy_brain_network/

4
https://www.chesapeakeirb.com/
